# Stochastic modeling of empirical time series of childhood infectious diseases data before and after mass vaccination

**DOI:** 10.1186/1742-7622-3-9

**Published:** 2006-08-08

**Authors:** Helen Trottier, Pierre Philippe, Roch Roy

**Affiliations:** 1 Department of Social & Preventive Medecine, University of Montréal, Montréal, Canada; 2 Department of Mathematics and Statistics, University of Montréal, Montréal, Canada; 3 Department of Oncology, Division of Cancer Epidemiology, McGill University, 546 Pine Avenue West, Montréal, Qc., Canada

## Abstract

The goal of this paper is to analyze the stochastic dynamics of childhood infectious disease time series. We present an univariate time series analysis of pertussis, mumps, measles and rubella based on Box-Jenkins or AutoRegressive Integrated Moving Average (ARIMA) modeling. The method, which enables the dependency structure embedded in time series data to be modeled, has potential research applications in studies of infectious disease dynamics. Canadian chronological series of pertussis, mumps, measles and rubella, before and after mass vaccination, are analyzed to characterize the statistical structure of these diseases. Despite the fact that these infectious diseases are biologically different, it is found that they are all represented by simple models with the same basic statistical structure. Aside from seasonal effects, the number of new cases is given by the incidence in the previous period and by periodically recurrent random factors. It is also shown that mass vaccination does not change this stochastic dependency. We conclude that the Box-Jenkins methodology does identify the collective pattern of the dynamics, but not the specifics of the diseases at the biological individual level.

## Background

Childhood infectious diseases that induce lasting immunity, such as measles, mumps and rubella are particularly well documented dynamically. Theoreticians have focused their attention on some of these infections because of the comparative simplicity of the processes that influence their transmission dynamics, the existence of long runs of data over many decades and also as a consequence of the remarkable periodicity of their incidences [1].

Modern theoretical epidemiology has been significantly influenced by the application of mathematics to the study of infectious diseases and by the development of the theoretical framework of the threshold theorem, according to which the introduction of a few infectious individuals into a community of susceptibles will not give rise to an epidemic outbreak unless the number of susceptibles is above a critical value [2]. An example of the expansion of this theorem was the development of the concept of a basic reproductive number (which defines the average number of secondary cases of infection generated by one primary case in a susceptible population). The above-mentioned assumptions enabled the development of systems of non-linear equations whose solutions describe the transmission dynamics in a population. The application of these explanatory models have provided insight into the transmission dynamics, such as the discovery of a critical community size for measles [3] or the critical level of mass vaccination required to block transmission within a defined community [4]. Essential insights into the persistence and stability of transmitted viral and bacterial infections that induce lasting immunity within large human communities was also gained by simple deterministic models (simulation) [5]. These mechanistic models, such as compartmental susceptible-exposed-immune-recovered (SEIR) models, predict the spread of infectious diseases, exhibit damped oscillations and mirror well the observed trends, such as for measles, which generally exhibits biennal cycles (with a seasonal pattern) before mass vaccination. The cycles are generated by the exhaustion of the supply of susceptibles through infection and its replenishment through new births, and may be perpetuated by seasonal changes in the rate of transmission, resulting from the aggregation and disaggregation of children for school terms and holiday periods. Cycles are more pronounced in infections with short infectious and latent periods, such as measles.

These simple mechanistic models (especially the seasonally-forced SEIR model) generate a rich array of dynamical behaviours, including chaotic patterns [6]. However, some authors have shown that the incorporation of more biological realism into the model (i.e., age structure), of realism in the seasonal forcing function, or of other spatial factors, can suppress the complex dynamic [7-10]. Adding biological complexity and heterogeneity generates a buffering effect that tends to reduce or eliminate the chaotic behaviour. The variety of dynamics seen in real populations with different demographic and geographic patterns suggests caution in the construction of models of complex systems. Thus, it is impossible to mimic the details of an epidemic on more than a qualitative level, even if these "explanatory" models are very useful for understanding the dynamics.

By contrast to simulation, empirical data on infectious diseases can be studied with a stochastic approach that models the dependent structure embedded in the time series. Box-Jenkins modeling, also called the Autoregressive Integrated Moving Average (ARIMA) method, seems promising for complementing infectious disease theories by describing the component structure ("non explanatory" analysis) of statistical time series. This method, widely used in the biological sciences, has rarely been applied to infectious disease modeling, despite the fact that it seems promising for many common infections that show seasonal behaviour and random changes or trends (non-stationary time series). Helfenstein [11] was the first to show that mumps and chickenpox time series from New York City (1928 to 1960) both have the same simple statistical structure. He concluded from his endeavour that apart from their clinical and epidemiological properties, it could be possible to classify the infectious diseases by the structure of their corresponding time series and that this might provide a better understanding of the disease. For example, he suggested that if the statistical structure of the time series for infectious diseases is different from that of non-infectious diseases, then this might provide insight into diseases such as multiple sclerosis or certain forms of leukemia for which an infectious agent is suspected but not established.

ARIMA models may also be relevant for the investigation of the relationship between different time series (for example, between the residuals of two series of diseases or between the residuals of a disease and those of an effector variable such as weather), to evaluate the impact of intervention (for example, the introduction of a screening test in the course of disease surveillance in a population or to measure the impact of an environmental catastrophe on a disease pattern) or to make forecasts of disease data. A few authors have used Box-Jenkins modeling to forecast the mortality incidence of pneumonia and influenza in the United States [12,13] or infectious diseases in Canada [14]. These authors were not really concerned with the possibility of evaluating the statistical structure of their disease time series.

In line with Helfenstein's (1986) contention [11], this paper will attempt to model time series of four childhood infectious diseases (pertussis, mumps, measles and rubella) using Box-Jenkins statistical methodology, in order to compare their specific statistical structure before and after mass vaccination in Canada. More specifically, we expect to correlate specific models either with vaccination phase, disease dynamics, or data quality. In other words, we attempt to characterize the basic statistical dynamics of these infectious diseases to obtain a better understanding of the different disease patterns, and explore the possibility of classifying the infections by their corresponding time series structure.

## Data

Data were obtained from Health Canada (Center for Infectious Diseases Prevention Control, Division of Disease Surveillance). Time series of pertussis, mumps, measles and rubella in Canada were available on a four-weekly basis (13 observations per year) before 1990 and on a monthly basis (12 observations per year) from 1991 to 2002, except for some periods where measles and rubella (1959–1968) and mumps (1959–1986) were not declared. The four-weekly and monthly time series were analyzed from the 1950s. In Canada, mass vaccination began in 1943 for pertussis, in 1963 for measles, and in 1969 for mumps and rubella (this could have varied for some provinces and territories). The study covered the pre- and post-vaccination era (except for pertussis where only the post-vaccination period is analyzed).

**Figure 1:**
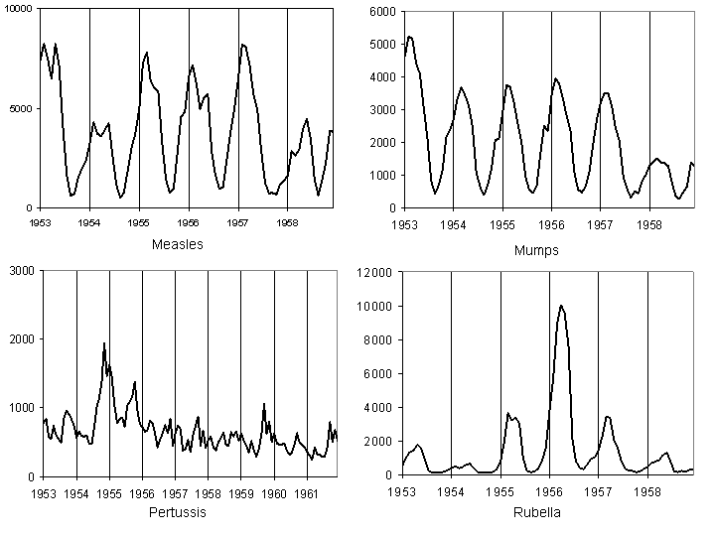
4-weekly notifications of measles, mumps, rubella (1953–1959) and pertussis (1953–1962) in Canada.

**Figure 2:**
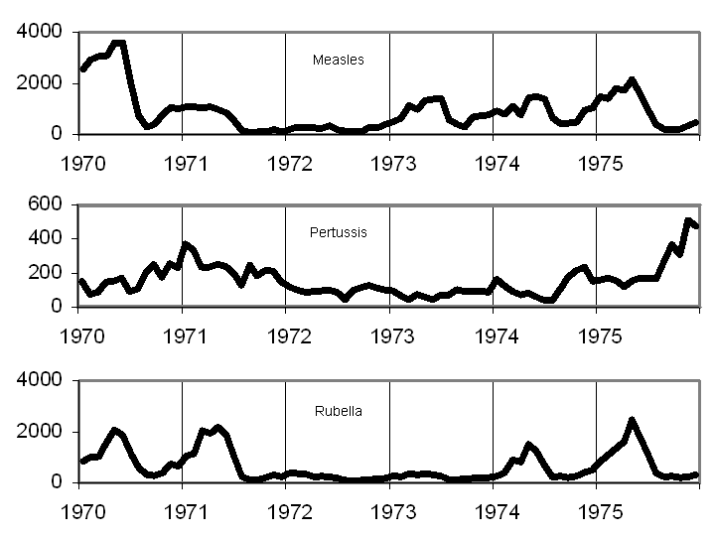
4-weekly notifications of measles, rubella and pertussis (1970–1976) in Canada.

**Figure 3:**
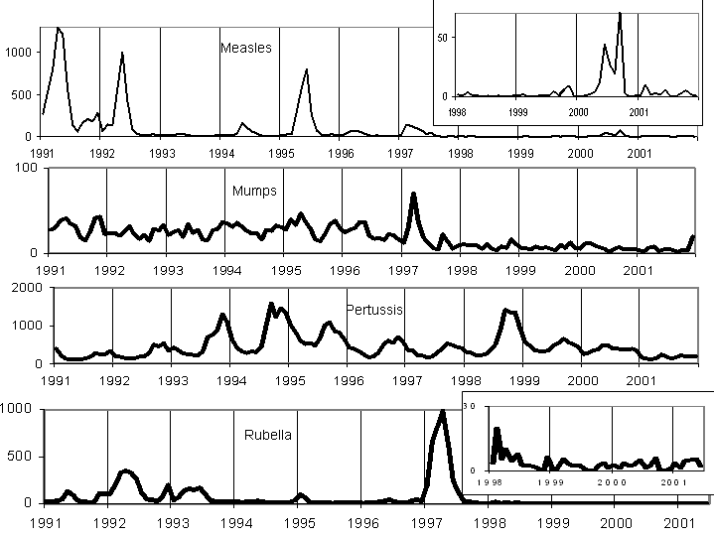
Monthly notifications of measles, mumps, rubella and pertussis (1991–2002) in Canada.

Notifications of infectious diseases on a four-weekly basis can be found in the *Annual Report of Notifiable Diseases *[15,16]. For the pre-vaccination era, four-weekly notification data from 1953 to 1959 for measles, mumps and rubella were analyzed. In the post-vaccination period, four-weekly notification data from 1970 to 1976 for measles and rubella were used (mumps was not declared from 1959 to 1986; it is therefore impossible to analyze data in the early post-vaccination era). In addition, four-weekly notifications of pertussis from 1953 to 1962 and from 1970 to 1976 were studied. Finally, to cover the current period, monthly notifications of measles, rubella and pertussis were used from 1991 to 2002 and mumps from 1991 to 1998 [17]. The crude time series for the three time periods are presented in figures 1 to 3.

Some four-weekly time series from 1970 to 1976 had unclassified annual data (i.e., without specifying in which of the four-weekly period of the year the cases appeared). For example, the pertussis time series had three unclassified observations in 1973. These data were proportionally distributed with respect to the percentage of cases that appeared in each four-weekly period. Since only a few cases were unclassified (<1% each year), we assumed that there was no effect on the dynamics. No other series analyzed (1950s and 1990s) had unclassified data. Further, monthly observations (1991–2002 data) do not have constant time intervals (they vary between 28 and 31 days). To control for this, we transformed the reported time series on a basis of 28 days. For example, the total number of cases that occurred in January was divided by 31 and multiplied by 28. This procedure allowed the cases to be equally spaced over a period of 28 days and allowed more comparable time series to be obtained. For time series that contained null values, we added the constant of one to the data. Monthly time series for measles (1991–2002) and rubella (1991–2002) were adjusted by adding a constant of one to all the data. Only 12 months (out of 144) for measles and six (out of 138) for rubella contained null values. This procedure did not change the temporal structure of the series, since one is added to all the data, and allowed us to use the logarithmic transformation.

Certain limitations, inherent in all notification data [18], must be considered. There are strong indications that not all cases come to the attention of physicians, which leads to underreporting of cases; further, the reporting of diagnosed cases is also considered incomplete [19-26]. Despite these shortcomings, the reporting system still reflects the dynamics (i.e., relative changes) quite accurately [15,16,19,27,28]. This type of under-reporting does not create a problem since our objective is to analyze the dynamics. Furthermore, the notification system in Canada has changed since 1950 (variation in the ways cases are reported, variation in case definition, consideration of laboratory confirmation, completeness of reporting) [15-17,19,25,26,29,30]. Also, the number of persons at risk may vary in the course of time. Had these phenomena been of some extent, they would be expected to cause problems by entailing inappropriate variations in the course of time. As stated above, the time series have been broken down into short sub-periods. This limited the extent of inappropriate variations in the data.

The choice of the series was conditioned by the changes in the surveillance system. The choice of the series was also limited by the fact that four-weekly time series were sometimes broken by a one-week notification period. For example, if the end-of-the-year notification period began on November 26 and ended on December 23 (28 days), the data between December 24 and 30 (7 days) were notified on a one-week basis in order to include the cases in the corresponding year. This procedure allowing adjustment of the data nevertheless caused problems by entailing non-constant time intervals in the series. The series have been selected with respect to these adjustments. Firstly, data before 1950 were excluded because some provinces and territories did not report the infectious diseases. The first time series thus cover the period from 1953 to 1959. In 1959, measles, rubella and mumps were excluded from the list of notifiable diseases in Canada. Pertussis was analyzed from 1953 to 1962. The time series was then truncated at the end of 1961, since the data were notified on a one-week period (to adjust for the data in the corresponding year). Measles and rubella notification were re-introduced in 1969. The second time series were selected from 1970 to 1976. In 1976, the series were truncated because the data were notified on a one-week rather than a four-week basis. It was not possible to analyze mumps data for this second period, as mumps was not notified between 1959 and 1986. The last series covered the period from 1991 to 2002. In 1991, the surveillance system was modified to introduce disease-specific case definitions by laboratory tests. The data were then declared on a month rather than four-week period.

## Methods

Box-Jenkins modeling requires a series of observations, obtained at equal time intervals on the same population. The following introduction is necessarily brief, but a more extensive introduction to this stochastic approach is available in many textbooks [31-35].

Denote the values of a series at equally spaced time *t*, *t*-1, *t*-2, ... by *Z*_*t*_, *Z*_*t*-1_, *Z*_*t*-2_... The Box-Jenkins method is based on the class of AutoRegressive Moving Average models (ARMA (*p, q*)) defined by the following equation:

*Z*_*t *_= *φ*_1 _*Z*_*t*-1 _+ ... + *φ*_*p *_*Z*_*t-p *_+ *a*_*t *_- *θ*_1 _*a*_*t*-1 _- ... -*θ*_*q *_*a*_*t-q *_+ *U*

where the *a*_*t*_'s are independent and identically distributed random shocks with mean 0 and variance σa2
 MathType@MTEF@5@5@+=feaafiart1ev1aaatCvAUfKttLearuWrP9MDH5MBPbIqV92AaeXatLxBI9gBaebbnrfifHhDYfgasaacH8akY=wiFfYdH8Gipec8Eeeu0xXdbba9frFj0=OqFfea0dXdd9vqai=hGuQ8kuc9pgc9s8qqaq=dirpe0xb9q8qiLsFr0=vr0=vr0dc8meaabaqaciaacaGaaeqabaqabeGadaaakeaaiiGacqWFdpWCdaqhaaWcbaGaemyyaegabaGaeGOmaidaaaaa@30E0@, *φ*_1_, ... , *φ*_*p *_and *θ*_1_, ..., *θ*_*q *_respectively denote the autoregressive and moving average coefficients and *U *is the model constant, which is related to the mean of the series. In such a model, the current time series observation *Z*_*t *_is explained by a linear combination of the *p *previous observations *Z*_*t*-1_, ..., *Z*_*t-p*_, a linear combination of the *q *previous random shocks *a*_*t*-1_,...,*a*_*t-q *_and the constant term *U*. The error term is represented by *a*_*t*_. If *q *= 0 we retrieve the class of pure autoregressive models of order *p *(AR(*p*)) and with *p *= 0 the class of pure moving average models of order *q *(MA(*q*)). Using the backward shift operator *B *such that *BZ*_*t *_= *Z*_*t*-1_, an ARMA (*p, q*) model can be represented in the more concise form:

*φ*(*B*)*Z*_*t *_= *U *+ *θ*(*B*)*a*_*t*_

where *φ*(*B*) = 1-*φ*_1_*B*- ... -*φ*_*p*_*B*^*p *^and *θ*(*B*) = 1 - *θ*_1_*B*- ... -*θ*_*q*_*B*^*q *^respectively represent the autoregressive and moving average operators. For stationarity, it is required that all the roots of the autoregressive operator lie outside the unit circle.

For adequate ARMA modeling, the time series must be stationary with respect to mean and variance. A simple and efficient way to achieve stationarity is to consider the difference between consecutive observations. Let ∇ = 1 - *B *denote the regular difference operator defined by ∇*Z*_*t *_= *Z*_*t *_- *Z*_*t*-1_. If a series has to be differenced to stabilize the mean, then the model corresponding to the original series is called AutoRegressive Integrated Moving Average (ARIMA). An ARIMA(p, d, q) model is defined by the equation:

*φ*(*B*)∇^*d*^*Z*_*t *_= *U *+ *θ*(*B*)*a*_*t*_.

In such a model, the *d*-th difference of the original series, ∇^*d*^*Z*_*t*_, is stationary and is represented by a stationary ARMA(*p, q*) model.

Most of the monthly, four-weekly or quarterly time series have a strong seasonal component (cyclical or periodic fluctuations that recur at the same phase of the cycle period). Indeed, some series may display seasonal non-stationarity that requires seasonal differencing. For a time series of period *s*, (*s *= 12 for monthly data and *s *= 13 for four-weekly data), stationarity is often achieved by working with the seasonally differenced series *Z*_*t *_- *Z*_*t-s*_. The seasonal difference operator of period *s *is usually denoted by ∇_*s *_= 1 - *B*^*s *^and a seasonal ARIMA model (SARIMA) is defined by an equation of the form:

φ(B)∇d∇sDZt=U+θ(B)at.
 MathType@MTEF@5@5@+=feaafiart1ev1aaatCvAUfKttLearuWrP9MDH5MBPbIqV92AaeXatLxBI9gBaebbnrfifHhDYfgasaacH8akY=wiFfYdH8Gipec8Eeeu0xXdbba9frFj0=OqFfea0dXdd9vqai=hGuQ8kuc9pgc9s8qqaq=dirpe0xb9q8qiLsFr0=vr0=vr0dc8meaabaqaciaacaGaaeqabaqabeGadaaakeaaiiGacqWFgpGzcqGGOaakcqWGcbGqcqGGPaqkcqGHhis0daahaaWcbeqaaiabdsgaKbaakiabgEGirpaaDaaaleaacqWGZbWCaeaacqWGebaraaGccqWGAbGwdaWgaaWcbaGaemiDaqhabeaakiabg2da9iabdwfavjabgUcaRiab=H7aXjabcIcaOiabdkeacjabcMcaPiabdggaHnaaBaaaleaacqWG0baDaeqaaOGaeiOla4caaa@46BB@

Usually, one seasonal difference is sufficient and *D *= 1 Often, stationarity is obtained by seasonal differencing. However, in some cases a regular difference may also be necessary if a trend remains in the data. In SARIMA models, the autoregressive and moving average operators are usually high order polynomials that involve terms in *B*^*s*^. In multiplicative models, these polynomials can be written as the product of a polynomial in *B *and of another one in *B*^*s*^. The polynomials in *B*^*s *^are called the seasonal components (SAR or SMA) of the model. The equations that define these models are given in most time series books, namely in [31,33,34].

Any successful modeling transforms time series into white noise (random residual series); a time series that does not require modeling (no trend and no dependent structure embedded) is white noise, i.e., a series of random shocks. One of the first steps in ARIMA modeling consists of stabilizing the variance of the series (stabilization of the mean is done by differencing). Various transformations like the square root or the logarithmic transformations can be appropriate. A practical tool for the choice of the appropriate transformation, based on the [36] power transformation, is the mean-range plot (the range of the data is plotted against the mean for each seasonal period) [11,37]. If the range is independent of the mean, no transformation is required. If the plot displays random scatter about a straight line, a logarithmic transformation is suggested. Other transformations can be used if a satisfactory model is not obtained by usual transformations.

ARIMA modeling is best done while following a protocol. The main steps required by Box-Jenkins modeling are model identification, parameter estimation and diagnosis. Identification is the key of model building. It allows specification of the model, i.e., identification of the number of regular and seasonal differencings and the order of the autoregressive and moving average operators.

The dependency structure of a stationary time series may be ascertained by the autocorrelation function (ACF). This function measures the correlation between *Z*_*t *_and *Z*_*t+k *_or, in other words, it is the correlation between a series and the same series lagged one, two, three... units. To help identify the process order, we also use the partial autocorrelation function (PACF). The PACF is the same as ACF except that the effect of the intervening observation(s) is removed. ACF and PACF patterns are generally recognized as belonging to certain types of ARIMA or SARIMA models. Minimizing either the residual variance, Akaike's information criterion (AIC) [38] or Schwarz's Bayesian Criterion (SBC) [39] helps identify the appropriate model. Once the model is specified, parameters of the model can be estimated. A rather straightforward iterative process is used. Diagnosis of the model consists of validating the model by examining its residuals. Residuals of a tentative model must be equivalent to white noise. Only random variations should be left in the residuals once main trends, dependencies, and cyclic variations have been removed from the series. ACF of the residuals and Ljung-Box statistics (global test for hypothesis of no correlation across a specified number of time lags) [40] are useful for testing the randomness of the residuals. In the case where ACF shows significant autocorrelations in the residuals, the protocol implies that one returns to the identification step of the protocol to explore the possibility of having missed an important component of the model or because of an unstable variance. If different models represent the data equally well, one chooses the simplest. An essential concept consists in finding a representation of the series that includes as few parameters as possible (this is the parsimony principle).

SAS version 9.1 was used to perform the analyses.

## Results

Mean-range plots were prepared for each infection time series. For example, from the mean-range plot for the pertussis time series (1953–1962, figure 4), it is seen that the square root transformation does not suffice to make the range independent from the mean, whereas the logarithmic transformation appears appropriate. Mean-range plots suggested the logarithmic transformation for every infection time series except rubella (1953–1959), for which a one over the square root transformation would perhaps be more appropriate (data not presented). Since the analyzed time series are short, it was not always clear which transformation was best; in this case we preferred to keep the same comparative base and to apply the logarithmic transformation for every time series, including the rubella case (1953–1959).

**Figure 4:**
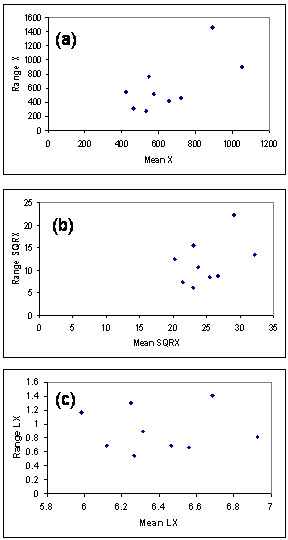
Mean-Range Plot for Pertussis (1953–1962). (a) Mean and range for non-transformed data (X). (b) Mean and range applying the square root transformation (SRX). (c) Mean and range applying the logarithmic transformation (LX).

**Figure 5:**
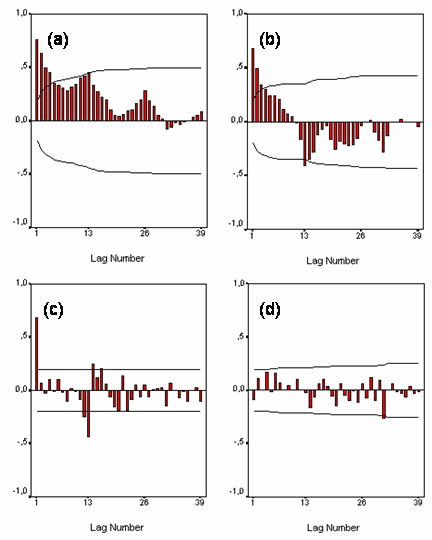
Pertussis (1953–1962): (a) autocorrelation function (ACF) of ln x_t_; (b) autocorrelation function (ACF) of ∇_13 _ln x_t_; (c) partial autocorrelation function (PACF) of ∇_13 _ln x_t_; (d) autocorrelation function of the residuals.

The following is a complete description of the analysis of the pertussis time series (1953–1962). The ACF of the logarithmically-transformed series (figure 5a) exhibits periodicity of length 13. This was expected since this childhood disease shows a seasonal cycle. Such an ACF pattern requires seasonal differencing (∇_13 _ln *Z*_*t *_= ln *Z*_*t *_- ln *Z*_*t*-13_) to induce seasonal stationarity. The periodicity disappears after seasonal differencing. The ACF and the PACF of ∇_13 _ln *Z*_*t *_in figures 5b and 5c allow one to identify a regular AR and a seasonal MA component. Indeed, the decaying ACF and the single peak at lag 1 in the PACF suggest an AR component of order 1. Also, the ACF and PACF give indications that a MA component with only a term of order 13 is appropriate since a sole peak at lag 13 is detected. Diagnostic checking shows that the series of residuals behaves as white noise. The residual ACF (figure 5d) exhibits no specific pattern and no marked peaks. This is confirmed by the Box-Ljung statistics (statistics *Q*), which gives lags 1, 13 and 26 P-values of 0.366, 0.643, and 0.579 respectively. It is therefore concluded that the error term in the estimated model is white noise and that the estimated SARIMA model fits the data best.

The following model has completely characterized the pertussis (1953–1962) time series:

(1-*φB*)∇_13 _ln *Z*_*t *_= *U *+ (1-Θ*B*^13^)*a*_*t*_

with parameter estimates (and standard errors) and residual variance:

φ^
 MathType@MTEF@5@5@+=feaafiart1ev1aaatCvAUfKttLearuWrP9MDH5MBPbIqV92AaeXatLxBI9gBaebbnrfifHhDYfgasaacH8akY=wiFfYdH8Gipec8Eeeu0xXdbba9frFj0=OqFfea0dXdd9vqai=hGuQ8kuc9pgc9s8qqaq=dirpe0xb9q8qiLsFr0=vr0=vr0dc8meaabaqaciaacaGaaeqabaqabeGadaaakeaaiiGacuWFgpGzgaqcaaaa@2E7C@ = 0.65 (0.07), Θ^
 MathType@MTEF@5@5@+=feaafiart1ev1aaatCvAUfKttLearuWrP9MDH5MBPbIqV92AaeXatLxBI9gBaebbnrfifHhDYfgasaacH8akY=wiFfYdH8Gipec8Eeeu0xXdbba9frFj0=OqFfea0dXdd9vqai=hGuQ8kuc9pgc9s8qqaq=dirpe0xb9q8qiLsFr0=vr0=vr0dc8meaabaqaciaacaGaaeqabaqabeGadaaakeaacuqHyoqugaqcaaaa@2E33@ = 0.84 (0.17), U^
 MathType@MTEF@5@5@+=feaafiart1ev1aaatCvAUfKttLearuWrP9MDH5MBPbIqV92AaeXatLxBI9gBaebbnrfifHhDYfgasaacH8akY=wiFfYdH8Gipec8Eeeu0xXdbba9frFj0=OqFfea0dXdd9vqai=hGuQ8kuc9pgc9s8qqaq=dirpe0xb9q8qiLsFr0=vr0=vr0dc8meaabaqaciaacaGaaeqabaqabeGadaaakeaacuWGvbqvgaqcaaaa@2DEF@ = -0.08 (0.02), σ^
 MathType@MTEF@5@5@+=feaafiart1ev1aaatCvAUfKttLearuWrP9MDH5MBPbIqV92AaeXatLxBI9gBaebbnrfifHhDYfgasaacH8akY=wiFfYdH8Gipec8Eeeu0xXdbba9frFj0=OqFfea0dXdd9vqai=hGuQ8kuc9pgc9s8qqaq=dirpe0xb9q8qiLsFr0=vr0=vr0dc8meaabaqaciaacaGaaeqabaqabeGadaaakeaaiiGacuWFdpWCgaqcaaaa@2E86@_*a *_= 0.216

The steps described for pertussis (1953–1962) analysis are not repeated here for the other time series since they lead to approximately the same type of model. A summary of the results is presented in table 1. For each time series, we provide *N*, the number of observations, the estimated parameters, the residual standard deviation (σ^
 MathType@MTEF@5@5@+=feaafiart1ev1aaatCvAUfKttLearuWrP9MDH5MBPbIqV92AaeXatLxBI9gBaebbnrfifHhDYfgasaacH8akY=wiFfYdH8Gipec8Eeeu0xXdbba9frFj0=OqFfea0dXdd9vqai=hGuQ8kuc9pgc9s8qqaq=dirpe0xb9q8qiLsFr0=vr0=vr0dc8meaabaqaciaacaGaaeqabaqabeGadaaakeaaiiGacuWFdpWCgaqcaaaa@2E86@_*a*_), the standard deviation of the transformed and differenced observed time series (σ^
 MathType@MTEF@5@5@+=feaafiart1ev1aaatCvAUfKttLearuWrP9MDH5MBPbIqV92AaeXatLxBI9gBaebbnrfifHhDYfgasaacH8akY=wiFfYdH8Gipec8Eeeu0xXdbba9frFj0=OqFfea0dXdd9vqai=hGuQ8kuc9pgc9s8qqaq=dirpe0xb9q8qiLsFr0=vr0=vr0dc8meaabaqaciaacaGaaeqabaqabeGadaaakeaaiiGacuWFdpWCgaqcaaaa@2E86@_*w*_), the coefficient of determination (*R*^2 ^= 1 - σ^
 MathType@MTEF@5@5@+=feaafiart1ev1aaatCvAUfKttLearuWrP9MDH5MBPbIqV92AaeXatLxBI9gBaebbnrfifHhDYfgasaacH8akY=wiFfYdH8Gipec8Eeeu0xXdbba9frFj0=OqFfea0dXdd9vqai=hGuQ8kuc9pgc9s8qqaq=dirpe0xb9q8qiLsFr0=vr0=vr0dc8meaabaqaciaacaGaaeqabaqabeGadaaakeaaiiGacuWFdpWCgaqcaaaa@2E86@^2^_*a*_/σ^
 MathType@MTEF@5@5@+=feaafiart1ev1aaatCvAUfKttLearuWrP9MDH5MBPbIqV92AaeXatLxBI9gBaebbnrfifHhDYfgasaacH8akY=wiFfYdH8Gipec8Eeeu0xXdbba9frFj0=OqFfea0dXdd9vqai=hGuQ8kuc9pgc9s8qqaq=dirpe0xb9q8qiLsFr0=vr0=vr0dc8meaabaqaciaacaGaaeqabaqabeGadaaakeaaiiGacuWFdpWCgaqcaaaa@2E86@^2^_*w*_). For each studied series, diagnostic checks based on the residuals failed to reveal any obvious inadequacy. The residuals of each series exhibit no specific pattern and no marked peaks. Furthermore, the Ljung-Box test does not reject the estimated model at the 5% significance level. Although the incidence of these four diseases has considerably decreased after the mass vaccination program (see fig. 1), the models remained similar. Comparison of the *R*^2 ^values indicates that except for mumps (1991–1998), the models explain between 68% and 90% of the variance of the differenced series. This is quite respectable in a time series context. All series exhibit an AR component of order 1 and a MA component of order 12 or 13. The rubella time series (1953–1959) and the measles time series (1970–1976) do not follow this model consistently, however. The major epidemic of rubella in 1956 (figure 1) as well as the one for measles in 1970 (figure 2) introduce significant variance in the time series and make it difficult to model. The best model that has characterized these series is:

**Table 1:** Estimates of parameters

**a) Pertussis**										
Period	N	AR (φ_1_)	AR (φ_2_)	AR (φ_6_)	SMA (Θ_12_)	SMA (Θ_13_)	Constant (U)	σ^ MathType@MTEF@5@5@+=feaafiart1ev1aaatCvAUfKttLearuWrP9MDH5MBPbIqV92AaeXatLxBI9gBaebbnrfifHhDYfgasaacH8akY=wiFfYdH8Gipec8Eeeu0xXdbba9frFj0=OqFfea0dXdd9vqai=hGuQ8kuc9pgc9s8qqaq=dirpe0xb9q8qiLsFr0=vr0=vr0dc8meaabaqaciaacaGaaeqabaqabeGadaaakeaaiiGacuWFdpWCgaqcaaaa@2E86@_w_	σ^ MathType@MTEF@5@5@+=feaafiart1ev1aaatCvAUfKttLearuWrP9MDH5MBPbIqV92AaeXatLxBI9gBaebbnrfifHhDYfgasaacH8akY=wiFfYdH8Gipec8Eeeu0xXdbba9frFj0=OqFfea0dXdd9vqai=hGuQ8kuc9pgc9s8qqaq=dirpe0xb9q8qiLsFr0=vr0=vr0dc8meaabaqaciaacaGaaeqabaqabeGadaaakeaaiiGacuWFdpWCgaqcaaaa@2E86@_a_	R^2^

1953–1962^1^	117	0.65*	-	-	-	0.84*	-0.08*	0.378	0.216	0.68
1970–1976^1^	78	0.89*	-	-	0.35	0.65*	0.08	0.693	0.296	0.82
1991–2002^2^	132	0.94*	-	-	0.58*	-	-0.05	0.569	0.213	0.86

**b) Mumps**										

Period	N	AR (φ_1_)	AR (φ_2_)	AR (φ_6_)	SMA (Θ_12_)	SMA (Θ_13_)	Constant (U)	σ^ MathType@MTEF@5@5@+=feaafiart1ev1aaatCvAUfKttLearuWrP9MDH5MBPbIqV92AaeXatLxBI9gBaebbnrfifHhDYfgasaacH8akY=wiFfYdH8Gipec8Eeeu0xXdbba9frFj0=OqFfea0dXdd9vqai=hGuQ8kuc9pgc9s8qqaq=dirpe0xb9q8qiLsFr0=vr0=vr0dc8meaabaqaciaacaGaaeqabaqabeGadaaakeaaiiGacuWFdpWCgaqcaaaa@2E86@_w_	σ^ MathType@MTEF@5@5@+=feaafiart1ev1aaatCvAUfKttLearuWrP9MDH5MBPbIqV92AaeXatLxBI9gBaebbnrfifHhDYfgasaacH8akY=wiFfYdH8Gipec8Eeeu0xXdbba9frFj0=OqFfea0dXdd9vqai=hGuQ8kuc9pgc9s8qqaq=dirpe0xb9q8qiLsFr0=vr0=vr0dc8meaabaqaciaacaGaaeqabaqabeGadaaakeaaiiGacuWFdpWCgaqcaaaa@2E86@_a_	R^2^

1953–1959^1^	78	0.82*	-	-	-	-	-0.17	0.335	0.195	0.89
1991–1998^2^	84	0.47*	-	-	-	0.54*	-0.08	0.406	0.365	0.20

**c) Measles**										

Period	N	AR (φ_1_)	AR (φ_2_)	AR (φ_6_)	SMA (Θ_12_)	SMA (Θ_13_)	Constant (U)	σ^ MathType@MTEF@5@5@+=feaafiart1ev1aaatCvAUfKttLearuWrP9MDH5MBPbIqV92AaeXatLxBI9gBaebbnrfifHhDYfgasaacH8akY=wiFfYdH8Gipec8Eeeu0xXdbba9frFj0=OqFfea0dXdd9vqai=hGuQ8kuc9pgc9s8qqaq=dirpe0xb9q8qiLsFr0=vr0=vr0dc8meaabaqaciaacaGaaeqabaqabeGadaaakeaaiiGacuWFdpWCgaqcaaaa@2E86@_w_	σ^ MathType@MTEF@5@5@+=feaafiart1ev1aaatCvAUfKttLearuWrP9MDH5MBPbIqV92AaeXatLxBI9gBaebbnrfifHhDYfgasaacH8akY=wiFfYdH8Gipec8Eeeu0xXdbba9frFj0=OqFfea0dXdd9vqai=hGuQ8kuc9pgc9s8qqaq=dirpe0xb9q8qiLsFr0=vr0=vr0dc8meaabaqaciaacaGaaeqabaqabeGadaaakeaaiiGacuWFdpWCgaqcaaaa@2E86@_a_	R^2^

1953–1959^1^	78	0.93*	-	-	0.31*	0.69	0.02	0.561	0.199	0.87
1970–1976^1–3^	78	0.63*	0.48*	-0.21*	-	-	-0.19	1.01	0.314	0.90
1991–2002^2^	132	0.78*	-	-	0.90*	-	-0.42*	1.676	0.834	0,75
**d) Rubella**										

Period	N	AR (φ_1_)	AR (φ_2_)	AR (φ_6_)	SMA (Θ_12_)	SMA (Θ_13_)	Constant (U)	σ^ MathType@MTEF@5@5@+=feaafiart1ev1aaatCvAUfKttLearuWrP9MDH5MBPbIqV92AaeXatLxBI9gBaebbnrfifHhDYfgasaacH8akY=wiFfYdH8Gipec8Eeeu0xXdbba9frFj0=OqFfea0dXdd9vqai=hGuQ8kuc9pgc9s8qqaq=dirpe0xb9q8qiLsFr0=vr0=vr0dc8meaabaqaciaacaGaaeqabaqabeGadaaakeaaiiGacuWFdpWCgaqcaaaa@2E86@_w_	σ^ MathType@MTEF@5@5@+=feaafiart1ev1aaatCvAUfKttLearuWrP9MDH5MBPbIqV92AaeXatLxBI9gBaebbnrfifHhDYfgasaacH8akY=wiFfYdH8Gipec8Eeeu0xXdbba9frFj0=OqFfea0dXdd9vqai=hGuQ8kuc9pgc9s8qqaq=dirpe0xb9q8qiLsFr0=vr0=vr0dc8meaabaqaciaacaGaaeqabaqabeGadaaakeaaiiGacuWFdpWCgaqcaaaa@2E86@_a_	R^2^

1953–1959^1–3^	78	1.27*	-0.38*	-	-	-	-0.05	1.000	0.358	0.87
1970–1976^1^	78	0,94*	-	-	-	0,82*	-0,08	0.816	0.265	0.89
1991–2002^2^	126	0,85*		-	0.90*	-0.10	-0,34*	1.664	0.711	0.82

* p < 0,05.

^1^four-weekly notifications.

^2^Monthly notifications.

^3^The rubella (1953–1959) and the measles (1970–1976) time series do not follow the SARIMA model (1,0,0)(0,1,1)_12 or 13 _consistently. See text for details.

(1-*φ*_1_*B*-*φ*_2_*B*-*φ*_6_*B*) ∇_13 _ln *Z*_*t *_= *U *+ (1-Θ_12_*B*^12^-Θ_13_*B*^13^) *a*_*t*_.

It includes an AR term of order 2 for both series as well as an AR term of order 6 for measles. With *φ*_2 _and *φ*_6 _= 0, this more complex model amounts to the one obtained for the other series.

## Discussion

The results show that time series of pertussis, mumps, measles and rubella have about the same stochastic dependence in their consecutive data. Despite the fact that these childhood infectious diseases are biologically different, generally the number of new cases in one period is given by the number of cases in the previous period and by periodically recurrent random shocks. In order to ease the discussion, AR-*h *(MA-*h*) denotes an AR parameter of order *h *(MA parameter of order *h*). An AR-1 term assumes that each observation is directly dependent (autocorrelated) on the past observation (incidence in April is, for example, dependent on incidence in March). Such a component shows that the dynamics of infectious diseases are not only related by random or exogenous factors, but also by factors that keep endogenous memory of the past. Also, MA-12 and/or MA-13 terms were usually found. The way that data are notified can explain the presence of the order of 12, 13 or both for the seasonal MA parameter. In effect, since four-week data in a specific year do not necessary refer to the same period in the next year, it could introduce small variations in the time series and harbour a small difference in the order of the seasonal MA component. A MA-12 or MA-13 term means that each value is dependent upon the current and past random shocks (random factors). Dependence on the previous-year random shock would mean that random factors have a carry-over effect on current-year occurrence of infectious diseases. Periodically recurrent random factors act in the same way over incidence variations. Temperature would be an example of a random factor that has such effect on infectious diseases.

Box-Jenkins modeling is based on the mathematical properties of the time series and not on the transmission dynamics of infectious diseases as alluded to previously. *Description *of the dependent structure of the time series does not necessarily enable *explaination *of its pattern. It is nevertheless possible that some factors account for the emergence of theses processes. In other words, the potential for an epidemic lies not only with the mean biological parameters of a disease, such as transmission risk or the duration of the infectivity or latent period, but just as well with the way society is organized, how often we travel, the size of our family, division of the school year, density of the population, etc. In the case of measles, for example, it is well known that the transmission potential is higher in autumn because of school entry [5]. Seasonal patterns of births are also well documented [41-44]. Moreover, existence of different viral strains magnifies the problem of elucidating the complex dynamics of infectious diseases. Future research, particularly in molecular epidemiology, could help understand these dependencies.

Our results suggest that childhood infections share some basic factors, and that mass vaccination does not seem to have changed the embedded stochastic dependency. In Canada, the pertussis vaccine was introduced in 1943, the measles vaccine in 1963, and the mumps and rubella vaccines in 1969. Unfortunately, national estimates of childhood vaccination coverage were not collected in a standardized manner or on a regular basis prior to 1994. In 1994 and 1998, annual surveys were conducted by the Division of Immunization, LCDC, to obtain population-based estimates of vaccination coverage of 2-year-olds in Canada [45]. Results of the 1998 survey show stable coverage levels compared to the previous 1994 surveys. The coverage levels for one dose of measles (96.0%), mumps (95.4%) and rubella (95.3%) vaccines are the highest for any of the routinely recommended vaccines and the closest to national targets for 97% coverage by the second birthday. Coverage levels for pertussis in the 1994 and 1998 surveys approximate 80% by the age of two years. Notwithstanding the large proportion of susceptibles vaccinated, vaccine failure can occur, so that a proportion remains susceptible. Higher immunity is reached for viral infection (measles, rubella, mumps). Thus, measles vaccine efficacy varies between 85% and 95% with a single dose of the vaccine given at 12 or 15 months and may reach 100% with a second dose [46-49]. A single dose of the mumps and rubella vaccines produces an antibody response in over 95% and 97% of susceptible individuals, respectively [48,49]. For pertussis (a bacterial infection), immunity might be lower. Efficacy of the pertussis vaccines was estimated to be no more than 85% and will not induce lasting immunity [48,50-52]. We therefore conclude that vaccination coverage and vaccine efficacy are relatively high for measles, rubella and mumps, but could be lower for pertussis.

Vaccination is the crucial public health intervention that limits epidemics by reducing the net case reproductive number (R) (number of transmissions/number of infected sources). If R is maintained below one, then the infection will be eliminated from the population. Among other interventions that have a bearing upon epidemics (to lower R) are isolation of infected cases from susceptibles during the infectious period, or administering antibiotics, which can reduce the infectious period of some diseases. Nevertheless, our analyses show that changes in these parameters do not affect the general statistical pattern of the epidemic.

**Figure 6:**
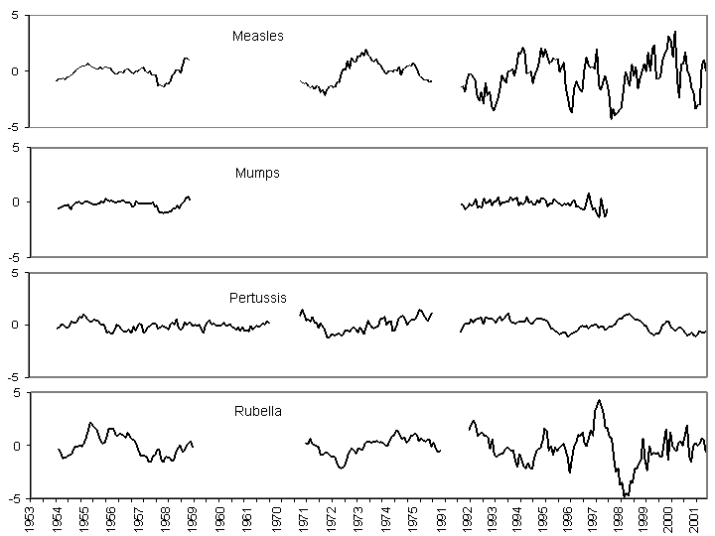
Seasonally differenced and logarithm transformed time series of 4-weekly (1953–1959, 1970–1976) and monthly (1991–2002) notifications of pertussis, mumps, measles and rubella in Canada.

Acknowledging the similar statistical structure of all our series remains a crude step, since the infection series differ in the magnitude of their AR and seasonal MA coefficients. However, interpretation of these coefficients must be done by keeping in mind that many biases in the notification data may affect the results [18,26]. Pertussis in the 1950s led to a lower AR-1 parameter (0.65) than in the 1970s and 1990s (around 0.90). That means that both series after 1970 are closer to the limit of non-stationarity and more dependent on preceding incidence. Comparing the seasonally differenced (∇_13 _ln *Z*_*t*_) series of pertussis for the three periods (figure 6), one sees that pertussis, in the 1970s and 1990s, shows more pronounced slow fluctuations than in the 1950s. Moreover, a greater impact of periodically-recurrent random factors seems to explain the dynamics of pertussis in the 1950s and 1970s. The seasonal MA parameter changes from 0.84 in the 1950s to 0.57 in the 1990s. It seems that variability in the 1950s is more random or subject to a few more random events. Finally, the deterministic linear trend in the 1950s, which is shown by the constant (p < 0.05) of the model, could be explained by mass immunization (which started in 1943 for pertussis).

Results for the mumps series should be interpreted with caution. For example, in some provinces, such as Québec, most of the recorded cases (>90%) are clinically-diagnosed, which can cause problems in terms of disease specificity [53]. Given the current high vaccination coverage and the low incidence of mumps, the positive predictive value of a diagnosis based solely on the presence of parotitis is probably low. Therefore, the reported number of clinically-diagnosed cases probably over-estimates true mumps occurrence [54]. The low positive predictive value is due to the low prevalence of mumps in an immunized population and to the fact that other viruses, including enteroviruses and influenza viruses, may also cause parotitis. Therefore, any overestimation of the number of mumps cases on the basis of the surveillance definition may have created problems for modeling, specifically for the 1990s. The AR-1 parameter for mumps was expected to be higher in the 1950s (0.82) and the 1990s (0.47). The New York mumps time series (from 1928 to 1960) exhibits an AR-1 of 0.93 and a MA-12 of 0.87 [11].

The estimated parameters of the measles and rubella series exhibit relatively constant results. However, the small trend in the seasonally-differenced rubella time series (1953–1959) suggested the application of regular differencing (figure 6). The model that best fits the regularly- and seasonally-differenced series has an AR-1 and a MA-13 term. Regular and seasonal differencing were also considered for the measles time series (1970–1976), which exhibits a significant epidemic peak in 1970. A model with an AR-1 term and a MA-13 term was also found. In those cases, replacement of the AR operator by a differencing operator ∇ = 1 - *B *(which corresponds to setting *φ*_1 _= 1) also leads to a particular case of the general model presented in table 1. A better model was searched and it came out that the seasonal differences of both series were best fitted by adding an AR-2 term. An AR-6 term was also required for the measles (1970–1976) series. However, we believe that those series should be inspected with caution since their variability is high.

Finally, from 1991 to 2002, the seasonally-differenced series of rubella (and measles) exhibit large fluctuations (figure 6). Childhood infectious disease time series generally exhibit highly significant seasonal and long-term cycles in pre-vaccination eras, but mass immunization reduces the relative importance of the cycles [5]. Seasonal oscillations of measles and rubella are less obvious in the 1990s and seasonal differencing may be less appropriate for these series. Also, these data could pose problems. It may occur that the large fluctuations alluded to above may have considerable importance, but be difficult to rescue by the Box-Jenkins methodology. The latter can appropriately *describe *regular patterns in data sets, such as linear trends, endemic data, or regular cycles of disease occurrence. It can even forecast data inasmuch as the pattern of past data is regular. The Box-Jenkins methodology can therefore perform best with infectious time series data that show regularly cycling epidemics unabated by either vaccination episodes or substantial changes in disease definitions that can profoundly disturb the required regularity of the series. On the other hand, simulations undertaken with a system of differential equations (the SEIR model, for instance), based on the biologic parameters of diseases (latent period, infectious period, reproductive rate, etc.), have been shown to mimic adequately, though qualitatively, irregular patterns of infectious diseases, such as unexpected epidemics of small and large sizes embedded in the same series. This may suggest that when it comes to irregular patterns, it is necessary to consider the non-linear dynamic mathematical basis of disease patterns, rather than to rely on the traditional linear statistical approach. Nonetheless, this does not mean that the Box-Jenkins methodology has had its last word, since the variation in the statistical parameters (AR, MA, etc.), which may bear significance, has not yet been paid sufficient attention.

In this paper, while considering Canada as a whole, space has been ignored. Clearly, spatial coupling of sub-populations decreases with distance (as temporal autocorrelations decay with time). However, given the robustness of our results we may confidentially assert that the conclusions would have remained the same had provinces been considered separately. Coupling can, however, be optimized in sub-populations analysis, that can remain undetected in large ensembles. Also, given large data sets and diagnoses of disease over, say, a regional area, coupling among local populations could bring up interactions and collective phenomena possibly detected by the Box-Jenkins methodology. Despite this, interaction among sub-populations would best be detected with a non-linear dynamic methodology that specially accounts for interactions. Further, since the results of the Box-Jenkins methodology are generally robust to ordinary fluctuations involving regular time series patterns, we expect that changes in the time scale of disease aggregation (from monthly to weekly or bi-weekly reports) would elicit no significant difference with respect to statistical modeling. Nevertheless, non-linear dynamic modeling might make a difference.

## Conclusion

Despite the fact that the studied infectious diseases are biologically different, it was found that they may generally be represented by simple models that have basically the similar statistical structure. The number of new cases in one month is given by the number of cases in the previous month and by periodically recurring random factors. Further, all series displayed seasonal drift that was accounted for by seasonal differencing. It was also shown that mass vaccination does not change this structural dependency. Even though mass vaccination was expected to have major impact on disease transmission dynamics (i.e., incidence, average age at infection, long-term periodicity, seasonal cycles), it does not clearly affect the stochastic dynamics. This statement should, however, be limited to the case with no breakdown of the disease regular pattern, either by mass vaccination or changes in disease definition. We therefore conclude that the Box-Jenkins stochastic modeling technique is generally robust to biological factors that are expected to entail significant effects in disease transmission dynamics. In cases of irregular patterns of disease, therefore, the Box-Jenkins method may fail, thus leaving the investigator with complex models that are genuinely difficult to interpret. Our initial attempt was to classify the series according to their stochastic dynamics; it came as a surprise that most series proved similar and mostly independent of the particular circumstances of the collective dynamics. Incidentally, it is highly significant that our models did not differ from those obtained by Helfenstein [11] despite our dealing with much shorter series and different diseases. Two things can be concluded from the above: (1) that the Box-Jenkins methodology does rescue regular patterns of disease, but modeling may fail when disturbed biological dynamics are at stake; (2) that the Box-Jenkins methodology is sensitive to the generic organizational principles of the disease population dynamics without reference to the individual biological parameters of the studied disease, i.e., it does identify the collective pattern of the dynamics, but not the specifics of the diseases at the biological individual level.

## Authors' contributions

HT was responsible for data collection, statistical analysis, results interpretation, and drafted the manuscript. PP was involved at each step of the analysis of the data; he contributed to the interpretation of the results, and supervised all of HT's work. RR was involved in the statistical and mathematical analysis and helped draft the manuscript.

## Competing interests

The author(s) declare that they have no competing interests.
